# Capacity for care: meta-ethnography of acute care nurses' experiences of the nurse-patient relationship

**DOI:** 10.1111/jan.12050

**Published:** 2012-11-19

**Authors:** Jackie Bridges, Caroline Nicholson, Jill Maben, Catherine Pope, Mary Flatley, Charlotte Wilkinson, Julienne Meyer, Maria Tziggili

**Affiliations:** Faculty of Health Sciences, University of SouthamptonUK; National Nursing Research Unit, King's College LondonUK; St Joseph's Hospice, LondonUK; School of Health Sciences, City University LondonUK; Barts Health, NHS TrustUKSchool of Health Sciences, City UniversityLondon, UK

**Keywords:** caring, experiences, hospitals, literature review, meta-ethnography, nurses, professional-patient relations, qualitative research, systematic review

## Abstract

**Aims:**

To synthesize evidence and knowledge from published research about nurses' experiences of nurse-patient relationships with adult patients in general, acute inpatient hospital settings.

**Background:**

While primary research on nurses' experiences has been reported, it has not been previously synthesized.

**Design:**

Meta-ethnography.

**Data sources:**

Published literature from Australia, Europe, and North America, written in English between January 1999–October 2009 was identified from databases: CINAHL, Medline, British Nursing Index and PsycINFO.

**Review methods:**

Qualitative studies describing nurses' experiences of the nurse-patient relationship in acute hospital settings were reviewed and synthesized using the meta-ethnographic method.

**Results:**

Sixteen primary studies (18 papers) were appraised as high quality and met the inclusion criteria. The findings show that while nurses aspire to develop therapeutic relationships with patients, the organizational setting at a unit level is strongly associated with nurses' capacity to build and sustain these relationships. The organizational conditions of critical care settings appear best suited to forming therapeutic relationships, while nurses working on general wards are more likely to report moral distress resulting from delivering unsatisfactory care. General ward nurses can then withdraw from attempting to emotionally engage with patients.

**Conclusion:**

The findings of this meta-ethnography draw together the evidence from several qualitative studies and articulate how the organizational setting at a unit level can strongly influence nurses' capacity to build and sustain therapeutic relationships with patients. Service improvements need to focus on how to optimize the organizational conditions that support nurses in their relational work with patients.

## Introduction

This article reports findings of a meta-ethnography of published qualitative research on nurses' experiences of nurse-patient relationships in acute settings. In a climate of increased demand on health services, of shifting professional roles and service reconfiguration, concerns are growing that the delivery of modern health care is lacking in compassion for patients and is failing to give the individualized care required by, for instance, older people with complex needs (Youngson 2008, Firth-Cozens & Cornwell 2009, Cornwell *et al*. 2012). Promoting meaningful connections with patients where practitioners see each patient ‘as a person to be *engaged with* rather than a body to *do things to*' (Nicholson *et al*. 2010, p.12) requires nurses and others to be able to articulate and appreciate the nature of these interactions and their impact on patient outcomes, along with an understanding of the factors that can promote or inhibit therapeutic relationships (Weinberg 2006). Nurses and nursing are now often portrayed as lacking in compassion and being distracted from these aspects of care (Corbin 2008, Flatley & Bridges 2008, Maben & Griffiths 2008). A range of high-profile reports in the UK into the quality of inpatient care for older people suggest that many of the reported problems centre on a lack of humanity in hospital staff (Department of Health 2010, Care Quality Commission 2011, Commission on Dignity in Care for Older People 2012). Other evidence suggests that these problems are relevant internationally (Bridges *et al*. 2012). It is also clear, however, that good practice does exist but we understand little about the conditions in which high quality, compassionate inpatient care is delivered. Insight into nurses' experiences as they engage with patients is therefore critical to understanding how best to support existing good practice and to focus service improvement initiatives. This focus is of particular importance in acute settings where patient throughput, service configuration and staffing patterns reduce contact time between staff and patients. In addition, while there is now a wealth of research findings on promoting nursing job satisfaction and motivation and reducing stress and burnout, we lack shared understanding about how nurse-patient relationships, the act of caring, and engagement in therapeutic relationships impact on nurses themselves.

There are an increasing number of primary qualitative studies relevant to this topic and these necessarily tend to rely on case study designs and smaller samples. A systematic overview of this work has not been previously conducted and it is difficult to draw generalizable conclusions for practice. This article uses the review and synthesis method of meta-ethnography to integrate findings from qualitative research studies focused on nurses' experiences of the nurse-patient relationship with adult patients in acute inpatient hospital settings.

## The review

### Aims

This meta-ethnography aims to give the deeper insight needed into nurse-patient relationships by synthesizing research that explores the experiences of nurses in these relationships. The objectives were:

- To understand how nurses characterize their relationships with adult patients in acute inpatient hospital settings.
- To understand the strategies that nurses use to build and sustain relationships with patients.
- To understand the impact on nurses for being in the nurse-patient relationship.
- To identify the factors that influence the relationships between nurses and patients.

The focus on adult patients reflected a wish to better understand the factors associated with reported care failures in adult settings.

### Design

Synthesis was conducted using the meta-ethnographic method described by Noblit and Hare (1988). Meta-ethnography is concerned with the translation of individual qualitative studies into one another, through the re-interpretation and transformation of their analytic and theoretical concepts (Noblit & Hare 1988, Britten *et al*. 2002, Pope *et al*. 2007).

### Search methods

Papers were identified by combining searches of electronic databases and hand searches of references lists of papers retrieved. Databases searched included CINAHL, Medline, British Nursing Index, and PsycINFO. Medical subject headings and free text searches related to nurses, acute health services, experience, and qualitative research were used (see supporting information file/Figure S1). To reflect relatively current experiences in nursing, searches were restricted to papers published between January 1999–October 2009. Funding constraints restricted the search to items published in English.

### Search outcome

Database searches resulted in 2133 hits (see supporting information file/Figure S2). Three papers already known to the authors were added that were not identified through database searches. Scanning the reference lists of all retrieved papers led to 11 further relevant papers being identified. After review of titles and abstracts and removal of duplicates (*n* = 75) 303 papers were retrieved for more detailed evaluation.

While the aim was not to produce an exhaustive search or comprehensive sample (Noblit & Hare 1988), systematic search procedures were used to ensure a final sample of items that were conceptually rich and potentially able to make an important contribution to the synthesis (Malpass *et al*. 2009). The inclusion and exclusion criteria were developed iteratively (and applied retrospectively where necessary) with these requirements in mind. For instance, as potential items were identified, it became clear from their heterogeneity that a clearer conceptualization was needed of what constituted an acute hospital inpatient setting. For the purposes of this synthesis, items were thus included that related to inpatient units/wards that give medical, surgical, and/or critical care therapies to adult patients with a goal of recovery and discharge. This excluded, for instance, studies based on rehabilitation or continuing care settings for older people, but included studies based on gerontological wards for acutely ill older people. The full text of all 303 retrieved papers was read and the inclusion and exclusion criteria applied (Table 1). Two hundred and forty-five papers that did not meet the inclusion criteria were excluded at this stage. A sample of 58 papers (54 studies) was thus obtained for quality appraisal.

**Table 1:** Inclusion and exclusion criteria

Include	Exclude
Used qualitative methods to explore experiences	Main focus not experiences of the nurse-patient relationship
Explored nurses' self-reported experiences of the nurse-patient relationship	Study related primarily to psychiatric care, primary or community care, or public health
Explored relationships with adult patients in an inpatient acute hospital setting	Findings included on experiences of other healthcare professionals (including midwives)
Reflected the perspectives of registered/licensed nurses (including licensed practical nurses and enrolled nurses)	Findings included on experiences with patients who were children or adolescents
	Findings included on experiences with relatives
	Findings included on experiences in settings that were not acute inpatient settings
	All data not gathered in Europe, North America or Australia
	Not qualitative research
	Not research
	Not published journal paper

### Quality appraisal

Each primary study was appraised using the Critical Appraisal Skills Programme (CASP) criteria for evaluating qualitative research (Critical Appraisal Skills Programme 2006) and was evaluated to appraise the degree to which they provided a rich account of participants' experiences of the nurse-patient relationship (Thomas & Harden 2008). Following the CASP appraisal, reviewers were asked ‘Taking into account your quality judgements above, what weight of evidence would you give this study in terms of whether its findings give a rich insight into nurses' personal lived experiences of being in the nurse-patient relationship – high, medium or low?’ This approach reflected a desire to include items that provided the conceptual richness needed for the meta-ethnography. Only studies judged as high ‘weight of evidence’ (WOE) were included in the final synthesis (see supporting information file/Table S1 for medium and low WOE studies). While there is no consensus in the meta-ethnography field to guide practice in using quality appraisal to inform selection, this decision is supported by analyses of two other syntheses of qualitative studies that concluded that synthesis findings were robust in the absence of lower quality studies, suggesting that they contribute little to the findings (Malpass *et al*. 2009, Bridges *et al*. 2010).

### Data abstraction and synthesis

Synthesis began with repeated readings of the studies to identify key categories and to determine relationships between individual studies. A list of key categories was thus generated and used as the basis for comparing and sorting interpretations, examining similarities and differences, and then integrating these in a new (‘third order’) interpretation that applies across the studies, referred to as a ‘line-of-argument’ (Noblit & Hare 1988, Britten *et al*. 2002, Pope *et al*. 2007). Britten *et al*. (2002) distinguish between different levels of interpretation, citing research participants' interpretations as ‘first order’, researchers' interpretations in the primary studies as ‘second order’, and the interpretation provided through a synthesis as ‘third order’. For this synthesis, second-order interpretations were extracted against the list of key categories identified and these were used as a foundation for exploring translations between the studies. Much of the detail of the second-order interpretation was retained at this stage, to help preserve context and meaning. Comparisons were then made across the studies to determine the extent to which concepts proposed in one study related to those expressed in another study, a process known as reciprocal translation (Noblit & Hare 1988). Differences were pursued as rigorously as similarities and comparisons across concepts and contexts were continuously made by, for instance, exploring the extent to which an emerging interpretation was relevant across clinical settings. The translated concepts were then used to identify third-order interpretations that transcended the individual accounts (Pope *et al*. 2007). As third-order interpretations emerged, they were systematically tested by looking across all the studies and the second-order interpretations; these third-order interpretations were discarded or developed further as required. Reciprocal translation continued until no further third-order interpretations emerged.

Two researchers [JB and MT] undertook database searching and preliminary study selection. Subsequent stages of quality appraisal, final selection, data extraction, and analysis were undertaken by a single researcher [JB]. To ensure alternative perspectives were advanced and discussed with a view to enriching the analyses, this was undertaken in consultation with a team of nurse action researchers conducting a project on dignity in care in two UK acute hospital trusts (http://www.city.ac.uk/dignityincare) and a researcher with expert knowledge of the nursing well-being literature. Organization of the review was managed through EPPI-Reviewer, an online software tool (Thomas & Brunton 2006) and synthesis with the aid of Microsoft Excel 2003 SP3. A protocol used to guide the review is available from the authors on request.

## Results

Of the 58 reports that met the inclusion criteria, 18 papers which reported 16 unique studies were graded as high quality and included in the synthesis. Summary information on the included studies is shown in Table 2. The studies were carried out in a range of countries and all the studies that specified the level of the included nurses focused on registered nurses with exception of one study on enrolled nurses. Eight studies were set in critical care. Six were set on general ward settings (medical, surgical, cancer, care for older people) and two studies included nurses from critical care and general ward settings. Twelve had a sample that included nurses with 10 or more years of nursing experience, one focused on newly qualified nurses, and three did not specify experience. The length of nursing experience of participants across the studies ranged from 2 months to 30 years. Ages ranged from 24–59 years. All the studies used qualitative interviews as the sole form of data collection.

**Table 2:** Summary information on selected studies (*n* = 16)

Study number	Summary reference (and country)
Critical care units	
1	Calvin *et al*. 2007
	The neuroscience ICU nurse's perceptions about end-of-life care (USA)
2	Gutierrez 2005
	Critical care nurses' perceptions of and responses to moral distress (USA)
3	Halcomb *et al*. 2004
	An insight into Australian nurses' experience of withdrawal/withholding of treatment in the ICU (Australia)
4	Hawley & Jensen 2007
	Making a difference in critical care nursing practice (Canada)
5	Hov *et al*. 2007
	Being an intensive care nurse related to questions of withholding or withdrawing curative treatment (Norway)
6	Kociszewski 2004
	Spiritual care: A phenomenologic study of critical care nurses (USA)
7	Söderberg *et al*. 1999
	Transforming desolation into consolation: the meaning of being in situations of ethical difficulty in intensive care (Sweden)
8	Wilkin & Slevin 2004
	The meaning of caring to nurses: an investigation into the nature of caring work in an intensive care unit (Ireland)
General wards	
9	Eriksson & Saveman 2002
	Nurses' experiences of abusive/non-abusive caring for demented patients in acute care settings (Sweden)
10	Hopkinson & Hallett 2002*,* Hopkinson *et al*. 2003
	Good death? An exploration of newly qualified nurses' understanding of good death Caring for dying people in hospital. (UK)
11	Mackintosh 2007
	Protecting the self: A descriptive qualitative exploration of how registered nurses cope with working in surgical areas (UK)
12	Nolan 2006, 2007
	Caring connections with older persons with dementia in an acute hospital setting–a hermeneutic interpretation of the staff nurse's experience Caring for people with dementia in the acute setting: a study of nurses' views (Ireland)
13	Nordam *et al*. 2005
	Ethical challenges in the care of older people and risk of being burned out among male nurses (Norway)
14	Quinn 2003
	Exploring nurses' experiences of supporting a cancer patient in their search for meaning (UK)
Critical care and general wards	
15	De Bal *et al*. 2006
	Involvement of nurses in caring for patients requesting euthanasia in Flanders (Belgium): A qualitative study (Belgium)
16	Kociszewski 2003
	A phenomenological pilot study of the nurses' experience providing spiritual care (USA)

The synthesis produced a line-of-argument which stated that nurses' capacity to build and sustain therapeutic relationships with patients is strongly influenced by the organizational conditions at a unit level; the organizational conditions in critical care units enhance nurses' capacity, while the conditions on general wards appear to inhibit nurses' capacity to build therapeutic nurse-patient relationships. This line-of-argument is illustrated through the third-order construct: influence of setting on capacity for caring and builds on three-second-order constructs identified through the synthesis (nurses' characterizations of relationships, relationship-building strategies, emotional impact on nurses). The second-order constructs are presented here first.

### Nurse-patient relationships (characterizations and strategies)

The synthesis findings enabled an overview of how nurses characterize their relationships with patients and the strategies they employ to build relationships with patients. Nurses in the individual studies consistently reflected characterizations of nurse-patient relationships as therapeutic or potentially therapeutic through the potential to support informed decision-making and treatment response assessment; to give the medium through which tailored care, comfort, and support is provided; to guide and support patient decision-making; to reconcile differing perspectives between patient, family, and professionals; and to act as patient advocate (Table 3).

**Table 3:** Nurses' characterizations of relationships with patients

Therapeutic or potentially therapeutic
Intimate knowledge of patient used to inform decision-making and assessing treatment responses (De Bal *et al*. 2006*,* Hawley & Jensen 2007)
Promotes dignity, comfort, emotional support, tailored holistic care (Quinn 2003*,* Wilkin & Slevin 2004)
Providing information, guidance and support to patient decision-making (Kociszewski 2003, Quinn 2003*,* De Bal *et al*. 2006*,* Hawley & Jensen 2007)
Reconciling perspectives between patients, families and clinicians (Calvin *et al*. 2007*,* Hov *et al*. 2007)
Being an advocate for patient (Söderberg *et al*. 1999*,* Kociszewski 2003, 2004*,* Gutierrez 2005*,* Nordam *et al*. 2005*,* De Bal *et al*. 2006*,* Nolan 2006, 2007*,* Calvin *et al*. 2007*,* Hawley & Jensen 2007)

In addition to nurses perceiving the relationship as therapeutic and as the medium for the delivery of high-quality care, the findings also reflected a range of strategies used by nurses to build relationships with patients. The studies consistently reflect that nurses aspire to make meaningful connections with patients, to gain a thorough knowledge of individual patients and their personal characteristics and to involve patients and families in a meaningful way in decisions made (Table 4). These aspirations for a therapeutic relationship held true across studies that include different clinical settings and nurses with varied professional experience:

**Table 4:** Nurses' strategies to build relationships with patients

Connecting with patients:

Unique position with patients and families because of prolonged contact (Quinn 2003*,* Halcomb *et al*. 2004*,* Gutierrez 2005*,* Hov *et al*. 2007)
Being ‘present’ in the relationship (Söderberg *et al*. 1999*,* Kociszewski 2003, 2004*,* Quinn 2003*,* Wilkin & Slevin 2004*,* Gutierrez 2005*,* Nordam *et al*. 2005*,* De Bal *et al*. 2006*,* Nolan 2006, 2007*,* Hawley & Jensen 2007)

Knowing the individual:

Nature of engagement enables nurse to get to know patient (Halcomb *et al*. 2004*,* Gutierrez 2005*,* De Bal *et al*. 2006*,* Hawley & Jensen 2007)
Intimate knowledge of the patient and family (Kociszewski 2003, 2004*,* Halcomb *et al*. 2004*,* Wilkin & Slevin 2004*,* Nordam *et al*. 2005*,* De Bal *et al*. 2006*,* Nolan 2006, 2007*,* Hawley & Jensen 2007)

Involving patients in their care:

Providing information, guidance and support to patient decision-making (Kociszewski 2003*,* Quinn 2003*,* De Bal *et al*. 2006*,* Hawley & Jensen 2007)

You can make a difference for the patient when you take into account what they are experiencing and perhaps what it means to be them. So I try to get as close as I can (Hawley & Jensen 2007, p.666)

#### Connecting with patients

Four of the studies reflect a perception by nurses that prolonged contact with individual patients and families through their 24 hour responsibility can place them in a unique position in the healthcare team, bearing witness to and alleviating the impact of illness and treatment and its meaning to individuals (Quinn 2003, Halcomb *et al*. 2004, Gutierrez 2005*,* Hov *et al*. 2007). Their perceived position ‘at the hub’ gives the potential to understand and play a key role in reconciling perspectives between patients, families, and other clinicians (Calvin *et al*. 2007, p.146, Hov *et al*. 2007). The connection between patients and nurses is perceived to be dependent on the nurse's ability to be ‘present’ in the relationship, that is to bring aspects of themselves to the relationship (rather than adopting a work persona), to expose themselves fully to the patient's and their own experiences, to be open and truthful in their dealings and to be generous in committing to the patient's best interests (Söderberg *et al*. 1999*,* Kociszewski 2004, Quinn 2003, Wilkin & Slevin 2004, Gutierrez 2005*,* Nordam *et al*. 2005, Nolan 2006, 2007*,* Hawley & Jensen 2007). Nurses perceive that connections of this kind enable them to promote dignity and to give comfort, emotional support, and holistic care that is tailored to what individual patients need (Quinn 2003*,* Wilkin & Slevin 2004).

#### Knowing the individual

The therapeutic potential of the relationship is based on an intimate knowledge of the patient and family, their illness and their coping strategies; and an appreciation of the importance to that individual of a range of psychological, social, environmental, and spiritual factors (Kociszewski 2003, 2004*,* Halcomb *et al*. 2004*,* Wilkin & Slevin 2004*,* Nordam *et al*. 2005*,* De Bal *et al*. 2006*,* Nolan 2006, 2007*,* Hawley & Jensen 2007). This knowledge of what is ‘salient, relevant, and qualitatively distinct in patient's particular situations’ (Hawley & Jensen 2007, p. 671) is seen by nurses as available to them through the nature of the engagement inherent in the nurse-patient relationship (Allsop & Saks 2002*,* Halcomb *et al*. 2004*,* Gutierrez 2005*,* Hawley & Jensen 2007). Nurses see the therapeutic benefit of this knowledge is its deployment in decision-making because it can inform ‘where the boundary between harm and benefit lies’ (Hawley & Jensen, p.667) and its deployment in assessment of an individual's response to treatment (De Bal *et al*. 2006*,* Hawley & Jensen 2007).

#### Involving patients in their care

The studies reflected that the perceived therapeutic potential of the relationship also lies in the nurse supporting the patient in making decisions congruent with patients' wishes and best interests (Kociszewski 2003*,* Quinn 2003*,* De Bal *et al*. 2006*,* Hawley & Jensen 2007). Nurses see their role as informing the patients about care principles and care alternatives, providing guidance in decision-making and supporting them in their search for meaning. They also perceive an important role for themselves in acting as intermediary in decision-making when there are conflicting views between the patient, the family, and the physician – a role that results from their unique understanding of the patient's particular situation (Calvin *et al*. 2007*,* Hov *et al*. 2007). The importance of honouring and advocating for the patient's choice emerged as key and reflects an aspiration for a decision-making process in which the wishes and interests of the patient and family are central:

Sometimes I feel really powerless and I do not have clear-cut answers, but I will not run away. I stay with the patient and we will see what will come (De Bal *et al*. 2006, p.593)

### Emotional impact on nurses

In addition to nurses' characterizations of and strategies for building nurse-patient relationships, several of the primary studies reported on the emotional impact on the nurse of being in the nurse-patient relationship. As Table 5 illustrates, the included studies reflect the strong feelings that are provoked by the nurse-patient relationship. If nurses are able to deliver care of a quality that matches their personal aspiration and that is seen as the best for that patient, they experience feelings of gratification, personal enrichment, and privilege:

**Table 5:** Emotional impact of relationship on nurses

Satisfaction

Delivering care matching aspirations leads to feelings of gratification, personal enrichment and privilege (Kociszewski 2003, 2004*,* Halcomb *et al*. 2004*,* De Bal *et al*. 2006*,* Nolan 2006, 2007*,* Calvin *et al*. 2007*,* Hov *et al*. 2007*,* Mackintosh 2007)

Distress

Contributing to unnecessary patient suffering – unable to relieve suffering, or implementing curative treatment plan with which they don't agree (Söderberg *et al*. 1999*,* Hopkinson *et al*. 2003*,* Halcomb *et al*. 2004*,* Gutierrez 2005*,* De Bal *et al*. 2006*,* Calvin *et al*. 2007*,* Hov *et al*. 2007)
Patient autonomy is constrained by factors outside of nurses' control (Eriksson & Saveman 2002*,* Nolan 2006, 2007)
Inadequate care (Eriksson & Saveman 2002*,* Nordam *et al*. 2005)

When the patient dies, you do feel a sense of loss. I enjoyed being a part of the process…You need and you want to be part of that experience (Calvin *et al*. 2007, p.145)

However, if nurses are not able to meet their aspirations, they experience guilt, regret, and frustration:

I heard he (the patient) had died earlier on the Sunday morning and I personally found that very difficult…hard that I hadn't told him he was dying, which he asked me to, I hadn't been there when he was dying, which I felt, I might have liked to have been, or to have some part of it and that my last interaction was, I was too busy to stop (Quinn 2003, p.169)

Findings suggest that there are particular patient groups that prompt greater distress. Patients who are dying prompt emotional distress in nurses as they bear witness to the suffering of patients and families (Hopkinson & Hallett 2002*,* Quinn 2003*,* Kociszewski 2004*,* Wilkin & Slevin 2004*,* Calvin *et al*. 2007*,* Hov *et al*. 2007*,* Mackintosh 2007) but moral distress can be triggered when nurses perceive that they are contributing to unnecessary additional suffering, either by implementing a treatment plan with a curative focus with which they do not agree, or because they are unable to relieve suffering because of factors outside of their control (Table 5). Caring for patients with dementia can also prompt moral distress where patient autonomy is constrained either by the physical environment or by the actions of nurses who lack the personal and organizational resources to deliver the care they would like (Table 5). Finally, caring for older people can prompt moral distress because of a lack of organizational capacity to give adequate care reflected in cooperation and communication difficulties with other professionals and higher patient throughput together with inadequate staffing levels (Table 5). Studies reflected that moral distress is closely linked with stress, burnout, and an emotional and physical withdrawal from working with particular patients and, in some cases, manifesting in a reluctance to be at work (Hopkinson *et al*. 2003*,* Gutierrez 2005*,* Nordam *et al*. 2005*,* Hov *et al*. 2007).

The findings from this synthesis affirm findings from individual primary studies that nurses perceive a therapeutic potential to the nurse-patient relationship and that the degree to which the relationship can be achieved can have a strong emotional impact on nurses. The synthesis has also enabled an analysis of influence of clinical setting on capacity for caring, leading to the development of a novel line-of-argument, reported on below.

### Influence of clinical setting on capacity to care

This final section of the findings introduces a novel line-of-argument, that nurses' capacity to build and sustain therapeutic relationships with patients is strongly influenced by the organizational conditions at unit level. This line-of-argument is illustrated through an analysis of the influence of setting on capacity for caring and builds on three-second-order constructs identified in the previous sections (nurses' characterizations of relationships, relationship-building strategies, emotional impact on nurses).

Studies reviewed reflected a range of factors perceived by nurses as influencing their ability to form a therapeutic relationship with patients, including the nurses' personal characteristics (experience, beliefs, personality, ability to talk openly) (Hopkinson & Hallett 2002*,* Kociszewski 2003, 2004*,* Quinn 2003, Halcomb *et al*. 2004*,* Nordam *et al*. 2005*,* De Bal *et al*. 2006*,* Calvin *et al*. 2007*,* Mackintosh 2007) and patients' personal characteristics (ability to communicate, dementia, agitation, aggression) (Wilkin & Slevin 2004*,* Nolan 2006, 2007*,* Mackintosh 2007), but organizational factors beyond the control of the individual nurse were the primary influence identified through the synthesis. As Table 6 illustrates, a clear contrast was identified between the perceptions of capacity of nurses in critical care settings and general settings, indicating that the nature of the clinical setting is a key determinant of nurses' capacity to build and sustain therapeutic relationships with patients.

**Table 6:** What does the synthesis add?

How does the clinical setting influence nurses' capacity for caring?

Critical care nurses frustrated that their intimate knowledge of the patient did not influence physician treatment plan (Halcomb *et al*. 2004*,* Gutierrez 2005*,* De Bal *et al*. 2006*,* Hov *et al*. 2007)
Critical care nurses more likely to report moral distress associated with contributing to unnecessary suffering (Söderberg *et al*. 1999*,* Hopkinson *et al*. 2003*,* Halcomb *et al*. 2004*,* Gutierrez 2005*,* De Bal *et al*. 2006*,* Calvin *et al*. 2007*,* Hov *et al*. 2007)
Nurses on general wards more likely to report frustrations in building and sustaining relationships (Söderberg *et al*. 1999*,* Eriksson & Saveman 2002*,* Hopkinson & Hallett 2002*,* Quinn 2003*,* Wilkin & Slevin 2004*,* Nordam *et al*. 2005*,* Nolan 2006, 2007*,* Mackintosh 2007)
Nurses on general wards more likely to report lack of time to build relationships (Söderberg *et al*. 1999*,* Eriksson & Saveman 2002*,* Hopkinson & Hallett 2002*,* Quinn 2003*,* Wilkin & Slevin 2004*,* Nordam *et al*. 2005*,* Mackintosh 2007*,* Nolan 2007)
Nurses on general wards report lack of organizational value attributed to building relationships (Eriksson & Saveman 2002*,* Nordam *et al*. 2005*,* Nolan 2006, 2007*,* Mackintosh 2007)
Nurses on general wards report moral distress associated with patient autonomy being constrained (Eriksson & Saveman 2002*,* Nolan 2006)
Nurses on general wards more likely to report active disengagement from nurse-patient relationship (see below) (Eriksson & Saveman 2002*,* Hopkinson & Hallett 2002*,* Nordam *et al*. 2005*,* Nolan 2006, 2007*,* Mackintosh 2007)

Disengagement from the nurse-patient relationship

Avoiding over-involvement with patients (Hopkinson *et al*. 2003*,* De Bal *et al*. 2006*,* Nolan 2006, 2007)
Reluctance to return to work (Gutierrez 2005)
Being a different person at work (Mackintosh 2007)
Avoiding certain patients and families (Gutierrez 2005)
Reluctance to care for patients at all (Gutierrez 2005)
Block out feelings/try to forget (Hov *et al*. 2007)
Frustrated aspirations lead to stress, burnout, patient abuse (Nordam *et al*. 2005)
Ignoring patients (Eriksson & Saveman 2002)

For nurses working in critical care settings, the most common issue reported related to the doctor's superior role in the team hierarchy (Halcomb *et al*. 2004*,* Gutierrez 2005*,* Hov *et al*. 2007). Nurses reflected that they do not always share the same goals for patient care that the doctors hold, with doctors often focusing solely on the curative aspects of treatment. Nurses saw their role as helping doctors understand what suffering and symptoms mean to individual patients and relatives but reported that doctors did not always accept nurses' judgements and overruled their views (Table 6). This issue reflects that critical care nurses often can and do form sufficiently close relationships with patients to feel able to act as their advocates in treatment decisions, but that the relationship with medical colleagues determined whether or not this advocacy role could be realized. Following a situation where a physician sited an intravenous cannula into a patient's arm against her clearly expressed wish, a nurse in Gutierrez's (2005) study reflects that not acting as the patient's advocate had a deleterious impact on her relationship with the patient:

It all happened very quickly. It wasn't until after the look crossed her face that I realized how violated she felt…It was a time when I should have been the patient's advocate and I wasn't on my toes, I didn't realize what was going on. And the loss of trust with that patient…in me. She looked at me when he left and wrote on her (communication) board ‘How could you let that happen?’ She never fully trusted me again after that…It's something you knew down here, in your gut. It was an awful loss (Gutierrez 2005, p.234)

In contrast, general ward nurses commonly reflected a lack of capacity to form therapeutic relationships with patients (Table 6). Key issues reported here were lack of time and a lack of organizational value attributed to nurse-patient relationships. These issues related to the level and acuity of nursing work coupled with inadequate staffing and appeared particularly associated with patients with complex needs such as older patients and patients with dementia:

What we lack is the possibility to sit down and to figure out, in a reasonable way, how to best help and treat the demented patient. But it can't be done here in an acute ward, we have our routines and everything is already fixed. We just have to carry on to make the work run as smoothly as possible. There isn't any time for solving conflicts. Instead you find yourself running away from them. Nor do we have the time to find out how to behave towards the demented person (Eriksson & Saveman 2002, p.82)

On the general wards, organizational value was attributed to maintaining ‘fixed’ ‘routines’ (Eriksson & Saveman 2002, p.82) at the expense of attending to complex patient needs:

Talking to patients is important. But there has to be opportunities to communicate and that is the problem. As a nurse, you feel ill at ease with that lack of time. You would like to spend some time with that patient, but you are hindered. It is a ‘lack of being’ instead of a lack of time. You aren't able *to* be there for your patient (De Bal *et al*. 2006, p.594)

This lack of support for caring activities appears linked with individual nurses choosing to not to employ the strategies identified as being required to build a therapeutic relationship (Table 4), but to employ instead strategies to actively disengage from the nurse-patient relationship to protect themselves (Table 6). The need to use these strategies is linked with a reduced capacity for caring and was more commonly reported in general ward settings. For instance, Mackintosh (2007) found that nurses working in surgical areas developed coping mechanisms as their professional experience grew, the most common of which was ‘ability to switch off’ (p.986). Nurses reported developing a work persona that included switching off/withdrawal, loss of caring beyond a certain acceptable level and depersonalization of individuals and situations (Mackintosh 2007):

I think it is like a plastic shield that you put up and I think if you stick at it long enough and you're in the job long enough, it becomes a natural way (Mackintosh 2007, p.986)

Other studies reflected this disengagement:

At the same time as we face the suffering we try to roll down our blinds. It is very brutal. If I am to cope with this and not distress myself, I have to forget it (Hov *et al*. 2007, p.207)

It's a good thing if you can make the patient take a sedative after lunch, then they'll hopefully sleep until the evening meal and I'll have time to do my job and report to the evening staff in peace and quiet (Eriksson & Saveman 2002, p.81)

Across the studies and regardless of setting, nurses described the main source of their emotional support as informal support from nursing colleagues (Hopkinson *et al*. 2003*,* Quinn 2003*,* Halcomb *et al*. 2004*,* Kociszewski 2004*,* Gutierrez 2005*,* Nordam *et al*. 2005). Few studies mentioned the existence of more formal support services and, where they did exist, nurses tended not to see them as helpful (Quinn 2003*,* Halcomb *et al*. 2004*,* Nordam *et al*. 2005).

In summary, the synthesis findings (summarized in Table 7) reflect that, while nurses share an aspiration for a therapeutic relationship with patients, the organizational setting at a unit level can strongly influence nurses' capacity to build and sustain such relationships. The findings also show that nurses working in organizational conditions, that inhibit their capacity to care, may then employ self-protection strategies which may further reduce their caring capacity.

**Table 7:** Synthesis, including second- and third-order interpretations

Categories	Second-order interpretations	Third-order interpretations
Nurses' characterizations of relationships with patients	(a) Relationships are therapeutic or potentially therapeutic to the patient;	
Nurses' strategies to build relationships with patients	(b) Nurses identify particular strategies that promote relationship: unique position, intimate knowledge, being ‘present’, nature of engagement;	(c) Some nurses use strategies to limit their emotional engagement with patients if their capacity to care is constrained by organizational conditions
Emotional impact of relationship on nurses Influencing factors	(d) Degree to which aspirations can be met dictates emotional impact: moral distress/satisfaction	(e) Organizational conditions at unit level strongly influence nurses' capacity to build and sustain therapeutic relationships

## Discussion

The aim of this meta-ethnography was to contribute to the debate about what nurses do and how best to support them in their work. Meta-ethnography is a systematic and rigorous method for synthesizing qualitative research which seeks to produce a conceptually rich account that is useful to policy makers, managers, and practitioners. Because it can produce novel third-order interpretations, it has greater value and generalizability than the individual studies on which it is based. Nevertheless, some limitations apply. Because of the intensive work involved in projects of this kind, there is a time-lag between the original database searches in October 2009 and publication. The studies included were limited to the experiences of registered or licensed nurses and so this synthesis gives no insight into relationships between patients and nursing support workers. In addition, all of the included studies reported findings based on interview data alone. The findings are thus limited to nurses' perceptions of their experiences and do not necessarily reflect what nurses actually do.

The synthesis identified three-second-order constructs (nurses' characterizations of relationships, relationship-building strategies and emotional impact on nurses) and one-third-order construct – the influence of setting on capacity for caring. The findings reflect that nurses aspire to an emotionally intimate therapeutic relationship with patients, that they attempt particular strategies to ensure that these relationships are therapeutic and that the degree to which their aspirations can be realized can have a strong emotional impact on nurses. These findings closely match the nursing mandate or contribution repeatedly advanced by and for the nursing profession over the past 25 years or so (Ersser 1991*,* Barber 1997*,* Dingwall & Allen 2001). They offer a reassuring message that counters concerns in the profession and among the general public that nurses are not as compassionate as they were in the past. These findings help us better understand that nurses also benefit from developing and sustaining therapeutic relationships with patients and this is an important finding in a context where negative emotions often attract greater attention (Dewar 2010). However, where nurses' aspirations are not achieved, they can experience distress and a desire to withdraw, either from caring for a particular patient, or from caring work altogether. Other empirical work has confirmed that there is often a difference between what nurses think they ought to be doing and what actually happens in practice and have linked this theory-practice gap with morale, job satisfaction and retention difficulties in nursing (Kramer 1974*,* Bendall 2006*,* Maben *et al*. 2006, 2007).

**What is already known about the topic**

- Nurses aspire to delivering therapeutic care through the medium of the nurse-patient relationship.
- The extent to which nurses are able to meet these aspirations has a strong emotional impact on nurses.
- Contemporary healthcare organizations may devalue healthcare activities that are not technical, physical, or codifiable.

**What this paper adds**

- The organizational setting at a unit level can strongly influence nurses' capacity to build and sustain therapeutic relationships with patients.
- The organizational conditions in critical care units enhance nurses' capacity to form therapeutic relationships better than the conditions on general wards.
- Some nurses deliberately limit their emotional engagement with patients if they do not feel supported in delivering high-quality care.

**Implications for practice and/or policy**

- Nurses need to better articulate the benefits to patients of the relational aspects of care.
- Acute care organizations and wider healthcare systems need to establish cultures that more visibly value and support therapeutic professional-patient relationships across organizations and at individual unit level and that reflect the emotional dimensions for all parties involved in healthcare delivery.
- Managers need to improve nurses' control over the conditions in which they work, optimize contact time between registered nurses and patients and ensure that clinical supervision and peer support is routinely available and accessible to all nursing staff.

Our unique contribution has been to identify through the meta-ethnographic method how the nature of the organizational setting at unit level can be a primary influencing factor on nurses' capacity to build and sustain therapeutic relationships with patients. The results show two clear organizational types, with nurses from general ward settings more frequently reflecting an impaired capacity to form therapeutic relationships with patients. The deliberate disengagement behaviours described for some general ward nurses contrast with the ideal of the nurse being ‘present’ in a relationship i.e. bringing self to the relationship and exposing oneself fully to the experiences in the relationship. They are associated with the distress inherent in nursing work and this links with findings from other studies that nurses can use a range of defensive strategies against the anxiety raised by the painful feelings invoked by nursing work (Menzies 1960*,* Allan 2001). But the meta-ethnography findings also illustrate that the disengagement behaviours result from the moral distress arising from an inability to give adequate care. Lack of time and an adherence to routine constrain general ward nurses' capacity to care. Williams *et al*. identified a key tension in acute care systems between ‘pace’ (the desire to discharge people as quickly as possible) and ‘complexity’ (taking account of the complex interaction between medical and social issues) (Williams 2001*,* Williams *et al*. 2009). While nurses have not relinquished direct control over nursing care, they are increasingly working in a managerialist environment with less autonomy over the conditions where care is delivered and where ‘pace’ dominates (Ackroyd & Bolton 1999*,* Adams *et al*. 2000*,* Williams *et al*. 2009). Nursing is then conceptualized as solely technical and physical work, while the more complex but less codifiable relational aspects of care are ignored or viewed as a ‘luxury’ by healthcare planners and managers (Dingwall & Allen 2001, p.65*,* Parker 2002*,* Maben 2008*,* Iles & Vaughan Smith 2009*,* Maben *et al*. 2010). The meta-ethnography findings indicate that the impact of these organizational conditions at a unit or ward level can result in moral distress for nurses because they cannot deliver the care they aspire to. Nurses then withdraw from attempting to emotionally engage with patients, having not received the support they need in the form of the right organizational conditions. We also found that, while nurses in critical care settings also have difficulty attaining their aspirations, especially as patient advocates, they do apparently have more capacity than nurses working on general wards to form therapeutic relationships with patients. Certain organizational conditions in critical care settings may help to explain the difference, for instance richer skill-mix and one-to-one (or one-to-two) nursing, both of which could enhance contact time between patients and nurses and thus capacity to care.

## Conclusion

The findings of this meta-ethnography reflect the importance of nurses and nursing openly acknowledging the complexity, struggle, and moral dilemmas inherent in nursing work. Nurses need to refocus current debate on the relational aspects of care, exploring and articulating their benefits and the conditions where they can be successfully delivered (Williams *et al*. 2009*,* Bridges *et al*. 2010). The findings that contrast nurses' experiences in critical care and general ward settings highlight the importance of unit-level conditions in shaping nursing work and indicate the conditions where relational work by nurses can flourish, although more research is needed to inform the development of suitable interventions. The nursing profession also needs to articulate how registered nurses can promote and best supervise relational care, when others, such as nursing assistants, may have more direct contact with patients. Other healthcare professions need to consider this review's findings and establish the relevance of them for their own practice. Acute care organizations and wider healthcare systems need to establish cultures that more visibly value and support therapeutic professional-patient relationships across organizations and at individual unit level and that reflect the emotional dimensions for all parties involved in healthcare delivery. Managers need to improve nurses' control over the conditions in which they work, optimize contact time between registered nurses and patients and ensure that clinical supervision and peer support is routinely available and accessible to all nursing staff, including nursing support workers.

We see the findings from this meta-ethnography as a contribution to an ongoing debate by nurses and nursing about what nurses do and as a resource for acute care organizations about how to support nurses in this work. The findings from this meta-ethnography make a contribution, through nurses' voices, to articulating the less visible aspects of nursing care in acute settings, but also the organizational conditions in which patients and nurses fare best.

## Supporting Information Online

**Figure S1.** Search strategy for Medline.

**Figure S2.** Study Selection Flow Diagram.

**Table S1.** Medium and low weight of evidence studies.
