# Supplementary material for: Capacity for care: meta-ethnography of acute care nurses' experiences of the nurse-patient relationship
Source: J Adv Nurs. 2012 Nov 19;69(4):760–72. doi: 10.1111/jan.12050 (PMC3617468; doi:10.1111/jan.12050)
Supplement: Supplementary file 1 [file jan0069-0760-SD1.docx]

**Supporting information file/figure 1**

**Search strategy for Medline**

1 (Nurse or Nurses or Nursing or Midwife or Midwives).mp. [mp=title, original title, abstract, name of substance word, subject heading word, unique identifier]

2 Nurses/ or Nursing Staff/ or Midwifery.mp. [mp=title, original title, abstract, name of substance word, subject heading word, unique identifier]

3 (Hospital or Emergency or Emergencies or Critical Care or Acute care).mp. [mp=title, original title, abstract, name of substance word, subject heading word, unique identifier]

4 Hospitals/ or Emergencies/ or Emergency Treatment/ or Critical Care/ or Inpatients/

5 (Experience or Experiences).mp. [mp=title, original title, abstract, name of substance word, subject heading word, unique identifier]

6 Qualitative.mp. [mp=title, original title, abstract, name of substance word, subject heading word, unique identifier]

7 Qualitative Research/ or Interview/

8 (Community or Public Health or Psychiatric or Psychiatry or Mental Health).mp. [mp=title, original title, abstract, name of substance word, subject heading word, unique identifier]

9 Public Health/ or Mental Health/ or Mental Health Services/ or Psychiatry/

10 1 or 2

11 4 or 3

12 6 or 7

13 8 or 9

14 5 and 10 and 11 and 12

15 14 not 13

16 limit 15 to (english language and yr="1999 - 2009")

17 limit 16 to (english language and yr="1999 -Current")
