# Supplementary material for: Capacity for care: meta-ethnography of acute care nurses' experiences of the nurse-patient relationship
Source: J Adv Nurs. 2012 Nov 19;69(4):760–72. doi: 10.1111/jan.12050 (PMC3617468; doi:10.1111/jan.12050)
Supplement: Supplementary file 2 [file jan0069-0760-SD2.docx]

**Supporting information file/figure 2**

**Study Selection Flow Diagram**

Records after duplicates (n=75) removed

(n=303)

Records excluded
(n =1769)

Full-text articles assessed for eligibility
(n = 303)

Studies included in meta-ethnography
(n= 18 papers reporting on 16 primary studies)

Full-text articles excluded, with reasons
(n = 285 )

Main focus not experiences (n=84)

Main focus not nurses (n=77)

Main focus not experiences with adult patients (n=53)

Main focus not acute in-patient setting

(n=46)

Not Europe, North America or Australia (n=12)

Not qualitative research (n=8)

Not research (n=7)

Not published journal paper (n=3)

Weight of evidence medium (n=23)

Weight of evidence low (n=17)

Additional records identified through other sources
(n =3)

Records identified through database searching
(n = 2133)

Records identified from the reference lists of full-text articles retrieved

(n=11)

Records screened

(n=2147)
