# Supplementary material for: Capacity for care: meta-ethnography of acute care nurses' experiences of the nurse-patient relationship
Source: J Adv Nurs. 2012 Nov 19;69(4):760–72. doi: 10.1111/jan.12050 (PMC3617468; doi:10.1111/jan.12050)
Supplement: Supplementary file 3 [file jan0069-0760-SD3.docx]

**Supporting information file/table 1**

**Medium and low weight of evidence studies**

Andersson EM, Hallberg IR and Edberg AK (2003) Nurses' experiences of the encounter with elderly patients in acute confusional state in orthopaedic care. *InternationalJournal of Nursing Studies* 40(4): 437-448

Aveyard H (2003) The patient who is unable to consent to nursing care. *International Journal of Nursing Studies* 40(7): 697-705

Aveyard H and Woolliams M (2006) In whose best interests? Nurses' experiences of the administration of sedation in general medical wards in England: An application of the critical incident technique. *International Journal of Nursing Studies* 43(8): 929-939

Badger JM (2005a) A descriptive study of coping strategies used by medical intensive care unit nurses during transitions from cure-to comfort-oriented care. *Heart & Lung: The Journal of Acute and Critical Care* 34(1): 63-68

Badger JM (2005b) Factors that enable or complicate end-of-life transitions in critical care. *American Journal of Critical Care* 14(6): 513-521

Barnard A, Hollingum C and Hartfiel B (2006) Going on a journey: understanding palliative care nursing. *International journal of palliative nursing* 12(1): 6

Botti M, Endacott R, Watts R, Cairns J, Lewis K and Kenny A (2006) Barriers in providing psychosocial support for patients with cancer. *Cancer Nursing* 29(4): 309

Bunch EH (2001) Hidden and emerging drama in a Norwegian critical care unit: ethical dilemmas in the context of ambiguity. *Nursing Ethics* 8(1): 57-67

Byers DC and France NEM (2009) The lived experience of registered nurses providing care to patients with dementia in the acute care setting: a phenomenological study. *International Journal of Human Caring* 12(4): 44-49

Cassidy I (2006) Student nurses’ experiences of caring for infectious patients in source isolation. A hermeneutic phenomenological study. *Journal of Clinical Nursing* 15(10): 1247-1256

Clarke A and Ross H (2006) Influences on nurses’ communications with older people at the end of life: perceptions and experiences of nurses working in palliative care and general medicine. *International Journal of Older People Nursing* 1(1): 34-43

Doherty C (2009) A qualitative study of health service reform on nurses' working lives: Learning from the UK National Health Service (NHS). *International Journal of Nursing Studies* 46(8): 1134-1142

Farnell S and Dawson D (2006) "It's not like the wards". Experiences of nurses new to critical care: A qualitative study. *International Journal of Nursing Studies* 43(3): 319-331

Goode D and Rowe K (2001) Perceptions and experiences of primary nursing in an ICU: a combined methods approach. *Intensive and Critical Care Nursing* 17(5): 294-303

Graham IW, Andrewes T and Clark L (2005) Mutual suffering: a nurse's story of caring for the living as they are dying. *International Journal of Nursing Practice* 11(6): 277-285

Hanks RG (2008) The lived experience of nursing advocacy. *Nursing Ethics* 15(4): 468-477

Henricson M, Berglund AL, Määttä S and Segesten K (2006) A transition from nurse to touch therapist—a study of preparation before giving tactile touch in an intensive care unit. *Intensive and Critical Care Nursing* 22(4): 239-245

Jezuit DL (2000) Suffering of critical care nurses with end-of-life decisions. *Medsurg Nurs* 9(3): 145-152

Mcgillis Hall L and Kiesners D (2005) A narrative approach to understanding the nursing work environment in Canada. *Social Science & Medicine* 61(12): 2482-2491

Mcmillen RE (2008) End of life decisions: nurses perceptions, feelings and experiences. *Intensive and Critical Care Nursing* 24(4): 251-259

Mohan S, Wilkes LM, Ogunsiji O and Walker A (2005) Caring for patients with cancer in non‐specialist wards: the nurse experience. *European Journal of Cancer Care* 14(3): 256-263

Nielsen B and Birkelund R (2009) Minority ethnic patients in the Danish healthcare system–a qualitative study of nurses’ experiences when meeting minority ethnic patients. *Scandinavian Journal of Caring Sciences* 23(3): 431-437

O’Connor T and Kelly B (2005) Bridging the gap: a study of general nurses’ perceptions of patient advocacy in Ireland. *Nursing Ethics* 12(5): 453-467

Ödling G, Norberg A and Danielson E (2002) Care of women with breast cancer on a surgical ward: nurses' opinions of the need for support for women, relatives and themselves. *Journal of Advanced Nursing* 39(1): 77-86

Peden-Mcalpine C and Clark N (2002) Early recognition of client status changes: the importance of time. *Dimensions of Critical Care Nursing* 21(4): 144-150

Roche-Fahy V and Dowling M (2009) Providing comfort to patients in their palliative care trajectory: experiences of female nurses working in an acute setting. *International Journal of Palliative Nursing* 15(3): 134-141

Ronayne C (2009) A phenomenological study to understand the experiences of nurses with regard to brainstem death. *Intensive and Critical Care Nursing* 25(2): 90-98

Rush KL, Robey‐Williams C, Patton LM, Chamberlain D, Bendyk H and Sparks T (2009) Patient falls: acute care nurses’ experiences. *Journal of Clinical Nursing* 18(3): 357-365

Searle C and Mcinerney F (2008) Creating comfort: nurses' perspectives on pressure care management in the last 48 hours of life. *Contemporary Nurse* 29(2): 147-158

Sherman DW (2000) Experiences of AIDS‐dedicated nurses in alleviating the stress of AIDS caregiving. *Journal of Advanced Nursing* 31(6): 1501-1508

Silén M, Tang PF, Wadensten B and Ahlström G (2008) Workplace distress and ethical dilemmas in neuroscience nursing. *Journal of Neuroscience Nursing* 40(4): 222

Skott C and Eriksson A (2005) Clinical caring–the diary of a nurse. *Journal of Clinical Nursing* 14(8): 916-921

Smallwood A and Humphreys M (2007) Nurses’ perceptions and experiences of initiating thrombolysis: a qualitative study. *Nursing in Critical Care* 12(3): 132-140

Smith C (2003) Researching the informal theories of nurses working with older people using a holistic, bio-psychosocial approach. *Quality in Ageing and Older Adults* 4(2): 36-47

Sumner J (2008) Is caring in nursing an impossible ideal for today's practicing nurse. *Nursing administration quarterly* 32(2): 92-101

Sundin-Huard D and Fahy K (1999) Moral distress, advocacy and burnout: theorizing the relationships. *International Journal of Nursing Practice* 5(1): 8-13

Taylor B, Bulmer B, Hill L, Luxford C, Mcfarlane J, Reed J and Stirling K (2002) Exploring idealism in palliative nursing care through reflective practice and action research. *International Journal of Palliative Nursing* 8(7): 324-330

Villanueva NE (1999) Experiences of critical care nurses caring for unresponsive patients. *Journal of Neuroscience Nursing* 31(4): 216-223

Vydelingum V (2006) Nurses’ experiences of caring for South Asian minority ethnic patients in a general hospital in England. *Nursing Inquiry* 13(1): 23-32

Wolf Z and Langner S (2000) The meaning of nursing practice in the stories and poems of nurses working in hospitals: a phenomenological study. *International Journal for Human Caring* 4(3): 7-17
